# L3 Skeletal Muscle Index Dynamics in Patients with HCV-Related Compensated Cirrhosis Following Sustained Virological Response after Direct Acting Antiviral Treatment

**DOI:** 10.3390/medicina57111226

**Published:** 2021-11-10

**Authors:** Florin Mihai, Anca Trifan, Carol Stanciu, Laura Huiban, Cristina Muzîca, Corina Lupașcu-Ursulescu, Dragoș Negru, Marius Lucian Savin, Irina Gîrleanu, Tudor Cuciureanu, Ana Maria Sîngeap

**Affiliations:** 1 Faculty of Medicine, “Grigore T. Popa” University of Medicine and Pharmacy, 700115 Iasi, Romania; florinmihai77@gmail.com (F.M.); stanciucarol@yahoo.com (C.S.); huiban.laura@yahoo.com (L.H.); lungu.christina@yahoo.com (C.M.); corina.ursulescu@gmail.com (C.L.-U.); draneg@gmail.com (D.N.); savinmarius@gmail.com (M.L.S.); gilda_iri25@yahoo.com (I.G.); drcuciureanutudor@gmail.com (T.C.); anamaria.singeap@yahoo.com (A.M.S.); 2 Department of Radiology, “St. Spiridon” Emergency Hospital, 700111 Iasi, Romania; 3 Institute of Gastroenterology and Hepatology, “St. Spiridon” Emergency Hospital, 700111 Iasi, Romania

**Keywords:** sarcopenia, skeletal muscle index, liver cirrhosis, sustained virological response, computed tomography

## Abstract

*Background and Objectives*: Sarcopenia is commonly associated with liver cirrhosis and predicts clinical outcome. Our aim was to identify the changes in skeletal muscle index (SMI) on computed tomography (CT) examination, as a quantitative marker of sarcopenia, in patients with HCV-related cirrhosis after direct acting antivirals (DAAs) treatment and to assess predictive factors for the evolution of SMI. *Materials and Methods:* This is a single center retrospective study in patients with HCV-related compensated cirrhosis who obtained sustained virological response (SVR) after DAAs. CT examinations were performed in 52 patients before and within 5–24 months after treatment. The total muscle area (*TMA*) of abdominal muscle at the level of third lumbar vertebra (L3) was measured at baseline and after SVR. The L3-SMI was calculated from *TMA* divided by body height squared (cm^2^/m^2^). We assessed changes in L3-SMI after SVR according to baseline body mass index (BMI) and laboratory data. Predictive factors were assessed by linear regression model. *Results:* Patients with L3-SMI above the gender-specific cut-off value at baseline had higher values of serum creatinine (median 0.73) compared to patients with low L3-SMI (median 0.68, *p* = 0.031). After SVR, 14 patients showed increase of L3-SMI, and 38 patients had a decrease of L3-SMI. BMI in the decreased L3-SMI group was significantly lower (median 26.17) than those without decreased L3-SMI (median 28.84, *p* = 0.021). ALT values in the decreased L3-SMI group (median 66.5) were significantly lower than those without a decrease in L3-SMI (median 88, *p* = 0.045). *Conclusions:* Low creatinine serum level correlates with sarcopenia. SMI was partially influenced by the viral clearance. Lower BMI and ALT serum levels at baseline were predictive for no benefit in terms of muscle mass dynamics. Understanding all the mechanisms involved in sarcopenia and identifying the most vulnerable patients could ensure optimal adapted care strategies.

## 1. Introduction

Sarcopenia is a syndrome characterized by progressive and generalized loss of skeletal muscle mass and impairment of muscular strength, which is commonly associated with various chronic pathologies, including chronic liver diseases [1]. In cirrhotic patients, sarcopenia is currently defined as the loss of muscle mass, and is a remarkably frequent abnormality ranging from 40–70% of that population [2,3] depending on the definition criteria, the type of study population, and the assessment methods. Sarcopenia is actually seen as a prevalent complication, which has been proved as a predictor for morbidity and mortality of cirrhotic patients [4,5].

The pathogenesis of sarcopenia basically results from an imbalance between protein synthesis and breakdown, but in cirrhosis, there are more complexes mechanisms, involving a particular skeletal muscle response and the anabolic resistance [6,7]. However, sarcopenia is considered a possibly modifiable condition, and proper interventions could have a favorable impact on patients’ prognosis. For these reasons routine assessment of sarcopenia is highly recommended, involving measurement of muscle mass, in addition to muscle strength and function.

Since the introduction of direct-acting antivirals (DAAs), the treatment of patients with HCV-related cirrhosis has been revolutionized. The main goal of treatment is to eliminate the virus by achieving a sustained virological response (SVR), lowering the risks of progression of the disease to liver failure and of hepatocellular carcinoma occurrence [8,9]. Alongside the aforementioned proven benefits of the viral clearance, there are hopes for the improvement of cirrhosis-associated comorbidities, such as sarcopenia.

Moreover, for cirrhotic patients treated with antiviral therapies, maintaining muscle mass is crucial and identifying pretreatment factors linked to the improvement in skeletal muscle mass is important in nutritional strategy.

Computed tomography (CT) is currently the most widely used method for evaluating sarcopenia [10,11]. It has the advantage of being widespread and therefore easy to access, it does not require special preparations of the patient before scanning, allowing the evaluation of muscle mass even on non-enhanced examinations. Multiple studies have considered different muscle groups, both thoracic and abdominal, to quantify muscle mass loss but the most widely used method uses total muscle area (*TMA*) at a section level passing through the third lumbar vertebra (L3) and allows the calculation of the skeletal muscle index (SMI) [11]. The L3-SMI is easy to assess and there are well-established cutoff values depending on gender [12]. L3-SMI is the preferred CT method of muscle mass assessment in the cirrhotic patient compared to the evaluation of other muscle surface areas such as the psoas that had a lower sensitivity in these patients [13].

The purpose of this study was to identify the changes in skeletal muscle mass, as quantitative marker of sarcopenia, in patients with HCV-related cirrhosis who achieved SVR after DAA treatment and to assess pretreatment predictive factors for the evolution of L3-SMI.

## 2. Materials and Methods

### 2.1. Study Design

This is a single center retrospective study in patients with genotype 1 HCV-related compensated cirrhosis who obtained SVR after treatment with DAAs. Eligibility criteria for antiviral therapy were those established at the time by the National Health Insurance Agency and recommended by the international guidelines: adult, experienced or treatment-naive patients with compensated Child-Pugh A cirrhosis. The main exclusion criteria were decompensated liver cirrhosis or evidence of hepatocellular carcinoma. Demographic and biological parameters were assessed for all patients at baseline.

In order to evaluate the dynamics of muscle mass, we included in the study only patients who had at least two CT evaluations: a CT examination before starting treatment with DAAs, and a CT examination after obtaining SVR. The interval between these two CT examinations was in a range from 5 to 24 months.

The study was conducted according to the guidelines of the Declaration of Helsinki and approved by the Ethics Committee of “Grigore T. Popa” University of Medicine and Pharmacy (number 15453, 23 May 2019).

### 2.2. Scanning Protocols

We used a Siemens Sensation^®^ 16 slice configuration CT scanner (Siemens AG Medical Solution, Erlangen, Germany) available in our institution. Patients were examined in the supine position, in post-inspiratory apnea. The scanning protocol includes a non-enhanced scan followed by intravenous administration of iodine-based contrast medium. The contrast medium was administered in bolus with an injection rate of 3–5 mL/s. The arterial phase was acquired at 30–35 s (late arterial phase) and the venous phase at 70–75 s after contrast injection.

### 2.3. Assessment of L3-SMI (Skeletal Muscle Index)

All imaging data were acquired from venous phase images using a single slice axial CT scan of the abdomen. The third lumbar vertebral body, with both transverse processes visible was chosen as a reference for the axial section utilized for processing.

**Figure 1:**
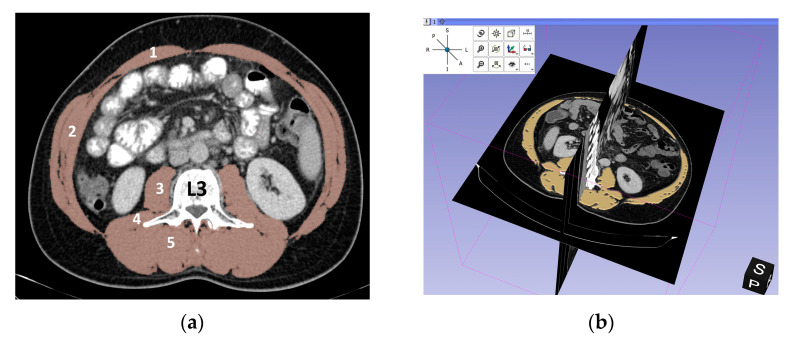
Axial cross-section at third lumbar vertebra (L3): (**a**) Total muscle area included: (1) rectus abdominis, (2) transversus abdominis, internal and external oblique, (3) psoas, (4) quadratus lumborum, (5) erector spine; (**b**) 3D representation of the axial section of interest at L3 level.

The designated axial section, with 3 mm thickness, was evaluated in the soft tissue window (WL 60 WW 360) and the truncal muscles were delimited. The included muscles are the psoas muscle, erector spinae, quadratus lumborum, transversus abdominis, external and internal obliques and rectus abdominis (Figure 1).

A semi-automated demarcation of the muscle tissue was based on Hounsfield unit (HU) thresholds from −29 to +150. We used for demarcation a 3D Slicer^®^ image computing platform, a free, open source and multi-platform software package used for medical and related imaging research [14]. If needed, manual corrections were applied by the reader.

The calculated total muscle area (*TMA*) was expressed in cm^2^. Skeletal muscle index (L3-SMI) was calculated dividing *TMA* to square height (*H*^2^) of patient expressed in m^2^ as follows:

$$SMI=\frac{TMA\left({\mathrm{cm}}^{2}\right)}{H^{2}\left(m^{2}\right)}$$

Low muscle mass was defined according to established *SMI* cut-offs for each sex: males: <52.4 cm^2^/m^2^, females: <38.5 cm^2^/m^2^ [10,11,15].

### 2.4. Statistical Analysis

The Shapiro–Wilk test was used to assess the normality of the distribution of the continuous variables. The differences between two independent groups for the continuous variable were assessed with the non-parametric Mann–Whitney U test, given the small sample size. For predicting changes in L3-SMI, candidate variables were identified by creating a linear regression model. Throughout the analysis a 95% confidence level was considered satisfying, setting the significance at *p* < 0.05.

## 3. Results

### 3.1. Baseline Data

Baseline characteristics of the patients included in our study are presented in Table 1. The study group included 52 patients (20 men and 32 women) with a median age of 59 years (range 42–79). L3-SMI in males at baseline ranged from 29.96 to 73.39 cm^2^/m^2^ (median 50.73 cm^2^/m^2^), whereas for women the baseline ranged from 21.74 to 60.52 cm^2^/m^2^ (median 37 cm^2^/m^2^). Considering the cut-off values specific for each sex, the overall percentage of patients with low L3-SMI value at baseline was 63.46% (33/52), with 80% (16/20) in the male group and 53.12% (17/32) in the female group.

### 3.2. Comparison of Baseline Characteristics between Patients with Low L3-SMI and Normal L3-SMI

Analysis of data began with testing for significant differences in baseline records between the group of patients who had low L3-SMI pretreatment and those who had normal L3-SMI values according with gender-specific cut-off values. The following parameters were evaluated: age, body mass index (BMI) and laboratory values for total bilirubin, serum albumin, platelets, total cholesterol, INR, alpha-fetoprotein, serum creatinine, AST and ALT. The test statistics table for baseline data are shown in Table 2.

The statistical analysis showed that there was a significant difference for the laboratory values of serum creatinine between the two groups. Patients with normal L3-SMI values had higher serum creatinine (median 0.73) compared to patients with low baseline L3-SMI (median 0.68, *p* = 0.031).

The other parameters investigated did not reveal significant differences between the two groups of patients at baseline.

### 3.3. Comparison of Baseline Characteristics between Patients with Decreased L3-SMI and with Increased L3-SMI Assessed after Sustained Virological Response (SVR)

To assess the dynamics of L3-SMI after treatment, the difference between L3-SMI values at baseline and those obtained after SVR was calculated. The differences were reported as a percentage of the initial value. The results were grouped in increased and decreased groups depending on the positive or negative values. In the studied group, the loss of muscle mass had a median of −7.5% (−1% to −28%) and in those with growth the median was 7.2% (0.9% to 34%).

After obtaining SVR, 14 patients showed an increase of L3-SMI, and 38 patients had a decrease in L3-SMI values.

To assess whether there are significant differences between patients with decreasing muscle mass (according to L3-SMI values) and those with increasing muscle mass with respect to the initial values of the collected parameters, we performed the Mann–Whitney U test. The results are summarized in Table 3.

The statistical analysis showed that BMI in the decreased L3-SMI group was significantly lower (median 26.17) than those without decreased L3-SMI (median 28.84, *p* = 0.021).

**Figure 2:**
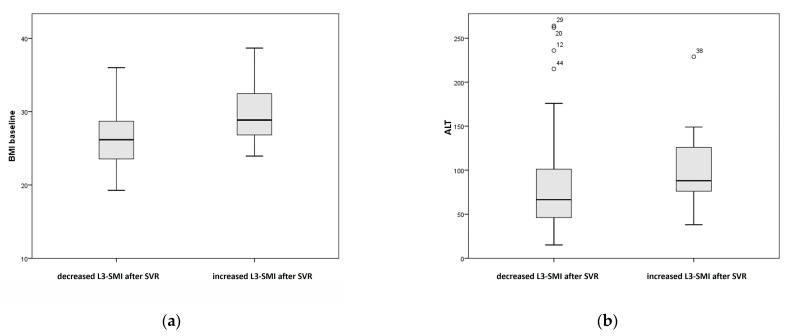
Boxplot for BMI (**a**) and ALT (**b**) values for patients with decreased L3-SMI and patients with increased L3-SMI after SVR.

Similarly, ALT values in the decreased L3-SMI group (median 66.5) were significantly lower than those without a decrease in L3-SMI (median 88, *p* = 0.045). Figure 2 shows the boxplots for the two variables analyzed.

### 3.4. Linear Regression Model

All parameters available at baseline were assessed as predictive factors in multiple linear regression and multiple logistic regression models. The only viable model was linear regression with a single predictor represented by BMI.

**Figure 3:**
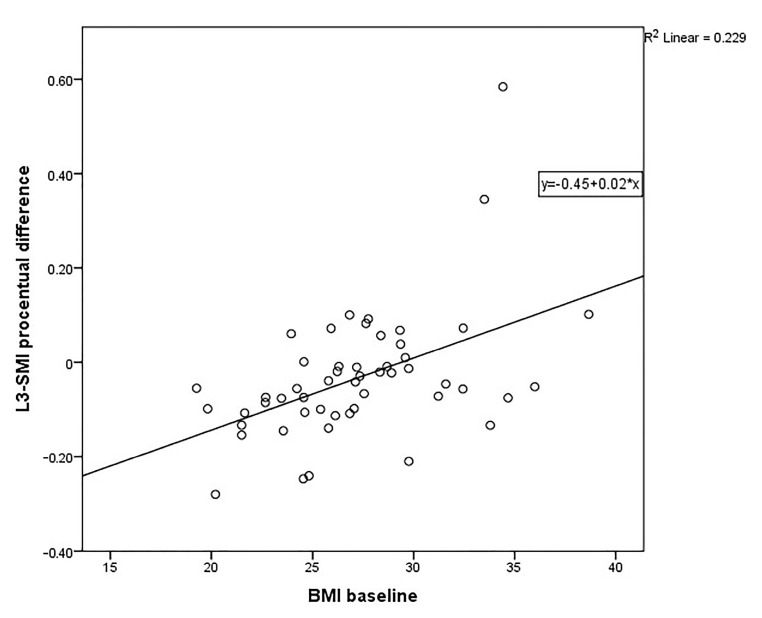
Linear regression for L3-SMI percentage change and BMI values at baseline.

Linear regression (Figure 3) targeting the percentage change in L3-SMI values after SVR showed significant prediction ability of the independent variable assessing the BMI at baseline (ANOVA F (1.50) = 14.83, sig = 0.00).

The variance of the single significant predictor obtained through the model, BMI at baseline, is able to predict at least 23% of the variance in the target variable L3-SMI (R2 = 0.229).

### 3.5. Comparison of Muscle Mass Dynamics after SVR between Groups with Low L3-SMI and Normal L3-SMI at Baseline

After treatment, 14 (28%) patients had an increase in L3-SMI values and 38 (72%) had a decrease in L3-SMI values.

Comparing the evolution of L3-SMI values after treatment according to baseline groups, normal L3-SMI and low L3-SMI, we found a statistically significant difference between patients with low L3-SMI values (median −1.3) and patients with normal L3-SMI (median −3.98, *p* = 0.02) (Table 4).

**Figure 4:**
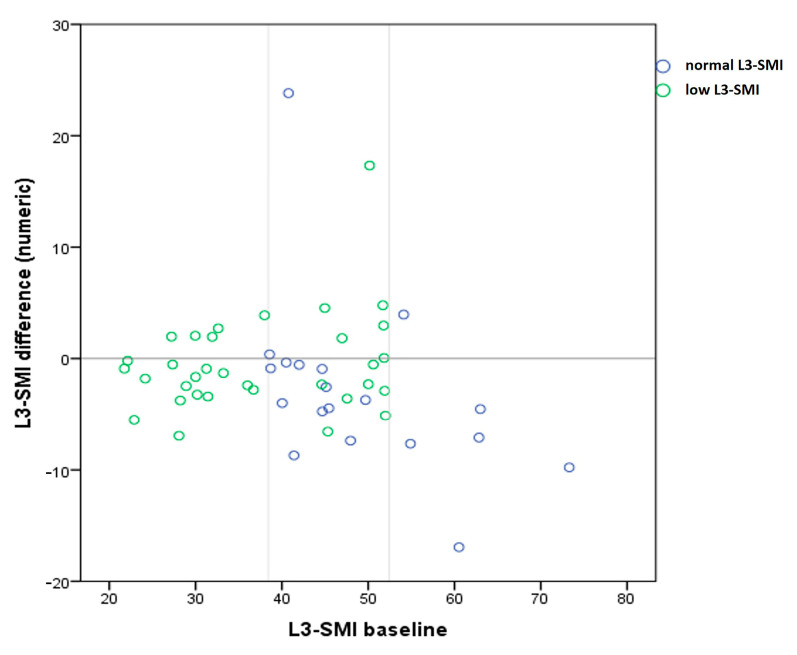
Scatter plot for L3-SMI difference after SVR in the baseline groups with low L3-SMI and normal L3-SMI. The difference in evolution is illustrated by the small number of patients in the group with initially normal values who showed an increase in L3-SMI values after SVR (above the zero line) compared to patients with low baseline values.

A higher number of patients in the sarcopenic group at baseline showed a post-SVR increase in L3-SMI values compared to the number of patients with normal baseline values. In patients with baseline low L3-SMI, 11 (33%) presented increased values after SVR while in patients with normal L3-SMI only 3 (16%) had an increase (Figure 4).

## 4. Discussion

Our study included patients with HCV-related compensated cirrhosis, treated with direct-acting antivirals. A comprehensive pre-treatment characterization of demographic and biological parameters was compiled, and CT evaluations were performed both at baseline and during follow-up after sustained virological response, aiming to identify changes in skeletal muscle mass status, eventually related to the improvement of liver function.

Analysis of baseline data showed the global predominance of patients with low L3-SMI (63.5% of all patients were underneath definition values, and median L3-SMI was inferior to cut-off values for both male and female patients’ groups), and proportion of patients with low L3-SMI values was higher in male patients compared to female patients (80% and 53.1%, respectively). Sarcopenia has been already identified as a frequent and important comorbidity related to liver cirrhosis [16] and its high prevalence in cirrhotic patients is not unexpected. So far, data showed that between 30% and 70% of cirrhotic patients are sarcopenic [6,17]. Nonetheless, because of its association with poor clinical outcome, such a high frequency of sarcopenia in a selected group of compensated cirrhosis must warn us about the importance of early nutritional care. A special consideration seems to be necessary in male patients, which appear to be more predisposed to sarcopenia, as previous data have already shown, both in the general population and in patients with liver cirrhosis [18,19,20].

Furthermore, analysis of baseline data showed a significant difference regarding serum creatinine levels according to L3-SMI values. Patients with low L3-SMI had decreased serum creatinine levels compared to patients with normal L3-SMI. Because serum creatinine is a classical biomarker reflecting the body’s muscle tissue mass [21], the results in our study are consistent with the current association between low creatinine levels and sarcopenia. No possible conditions that could have theoretically interfered with serum creatinine level dosage leading to falsely low values, such as advanced liver disease, fluid overload, and augmented renal clearance [22], were identified in our study, consequently low serum creatinine levels found in the low L3-SMI group of patients are interpreted as a direct image of their poor muscle mass status.

To assess the dynamics of muscle mass status following antiviral treatment, we compared L3-SMI results before and after SVR. Our analysis showed that, globally, most patients (72%) presented a decrease of L3-SMI values, while only a few more than a quarter (28%) obtained an increase of L3-SMI values. According to baseline muscle mass status, more patients with initial low L3-SMI presented an increased index after SVR, compared to patients with normal muscular status at baseline (33% vs 16%, respectively). Therefore, even if a benefit in terms of muscle mass was noted only in a minority of all patients, improvements were more frequent in patients who were most in need of muscle mass status correction. It should be noted that although most patients in the baseline group with normal L3-SMI values showed a decrease after treatment, only three patients fell below the threshold values, the rest remaining within the normal range. Undoubtedly, muscle mass dynamics has multiple underlying mechanisms, and sarcopenia in compensated cirrhosis is not completely decoded yet. Even if recent evidence suggests an increase or no more loss of skeletal muscle mass after antiviral treatment [23,24], the muscular improvement cannot be guaranteed solely by the viral clearance. As our results show, sarcopenia appears not to be solved only by the viral clearance, and supplementary nutritional interventions are necessary.

In our analysis, low BMI and low ALT were correlated to the decrease of L3-SMI after SVR. Classically, BMI is an indicator of nutritional status, and a higher BMI should consequently implicate a lower risk of sarcopenia. Even if sarcopenia is not excluded in overweight or even obese subjects, current thresholds showed lower sarcopenia prevalence with increases BMI [25]. Therefore, all efforts to counteract underweight and malnutrition must be made. ALT (alanine aminotransferase) is found in plasma and in various body tissue, being the most common in liver. Increased ALT is used as a biomarker for hepatocellular injury, while there is limited evidence about the clinical implications associated with low ALT levels. Even if ALT is traditionally part of liver function tests, its levels do not reflect liver disease severity, being dually linked to liver function status—usually increased in most liver injury, with possible normal results in advanced liver disease [26]. Several studies so far have demonstrated low serum ALT levels as predictive for long-term all-cause mortality among adults [27,28], while a recent study on patients with non-small lung cancer suggested a potential relation between low ALT serum activity and low muscle mass with increased frailty [29]. Thus, ALT has gained recognition as surrogate marker of frailty and shortened survival. In our study, lower ALT levels were predictive for the lack of benefit regarding muscle mass dynamics after SVR. In our relatively homogenous study group based on stage of liver disease, where all our patients had compensated cirrhosis, lower ALT levels appear to have predictive power concerning sarcopenia.

Our study has a few strengths and several limitations. As far as we know, this study is the first to discuss the CT evaluation of L3-SMI dynamics in cirrhotic patients who obtained SVR after DAAs treatment. As limitations, we could mention the relatively small number of patients included and the variable CT scan follow-up interval, due to the retrospective type of the study.

## 5. Conclusions

Sarcopenia, a frequent comorbidity in patients with advanced liver disease, can be objectively assessed by CT-based measurements. Even if the skeletal mass index is partially influenced by the viral clearance, some improvements were recorded in patients with baseline sarcopenia. Low creatinine serum level correlates with sarcopenia. Lower BMI and ALT serum levels at baseline were predictive for no benefit in terms of muscle mass dynamics. Understanding all the mechanisms involved in sarcopenia and identifying the most vulnerable cirrhotic patients could ensure optimal adapted care strategies.

## Figures and Tables

**Table 1:** Baseline characteristics of the patients.

Variables	Count or Median (Range)
Age (years)	59 (42–79)
Gender, male/female	20/32
Body mass index (kg/m^2^)	26.94 (19.27–38.67)
Skeletal muscle index (cm^2^/m^2^), male	50.37 (29.96–73.30)
Skeletal muscle index (cm^2^/m^2^), female	37.00 (21.74 -60.52)
Total bilirubin (mg/dL)	1.09 (0.43–2.47)
Serum albumin (g/dL)	3.91 (2.28–5.25)
Platelets ×10^4/mm^3^	10.9 (3.7–29.3)
Total cholesterol (mg/dL)	151.5 (80–221)
INR	1.14 (0.95–2.27)
Alpha-fetoprotein (ng/mL)	12.92 (2.14–131)
Serum creatinine (mg/dL)	0.72 (0.4–1.06)
AST (IU/L)	79 (28–224)
ALT (IU/L)	72 (15–264)

INR, international normalized ratio; AST, aspartate aminotransferase; ALT, alanine aminotransferase.

**Table 2:** Comparison between patients with low L3-SMI and normal L3-SMI.

	Normal L3-SMI (*n* = 19)		Low L3-SMI (*n* = 33)		Mann-Whitney	
	Median (Range)	Mean Rank	Median (Range)	Mean Rank	U	*p*
Age (years)	55.6 (42–73)	22.58	60 (42–79)	28.76	239.000	0.156
BMI	28.32 (20.2–34.42)	30.16	26.82 (19.27–38.67)	24.39	244.000	0.187
Total bilirubin	0.99 (0.6 -2.3)	24.29	1.16 (0.43–2.47)	27.77	271.500	0.425
Serum albumin	3.9 (2.49–5.25)	26.79	3.94 (2.28–4.75)	26.33	308.000	0.917
Platelets	10.6 (5.6–29.3)	26.76	10.9 (3.7–26.10)	26.35	308.500	0.924
Total cholesterol	148 (98–221)	23.61	158 (80–212)	28.17	258.500	0.296
INR	1.2 (0.95–1.55)	29.71	1.12 (0.97–2.27)	24.65	252.500	0.246
Alpha-fetoprotein	10.15 (3.82–131)	25.71	13.06 (2.14–110)	26.95	298.500	0.776
Serum creatinine	0.73 (0.53–1.06)	29.94	0.68 (0.4–0.92)	20.53	200.000	0.031 *
AST	84 (28–197)	25.34	78 (35–224)	27.17	291.500	0.676
ALT	68 (33–264)	27.74	75 (15–229)	25.79	290.000	0.655

INR, international normalized ratio; AST, aspartate aminotransferase; ALT, alanine aminotransferase, * significant.

**Table 3:** Comparison between patients with decreased L3-SMI and increased L3-SMI.

	Decreased (*n* = 38)		Increased (*n* = 14)		Mann-Whitney	
	Median (Range)	Mean Rank	Median (Range)	Mean Rank	U	*p*
Age (years)	59.5 (42–79)	27.04	57.07 (45–69)	25.04	245.500	0.672
BMI	26.17 (19.27–36)	23.55	28.84 (23.94–38.67)	34.50	154.000	0.021 *
Total bilirubin	1.1 (0.48–2.47)	26.71	1.0 (0.43–2.4)	25.93	258.000	0.869
Serum albumin	4.04 (2.28–5.25)	28.79	3.69 (2.44–4.5)	20.29	179.000	0.073
Platelets	11.6 (5.6–29.3)	27.20	10.25 (3.7–17.5)	24.61	239.500	0.584
Total cholesterol	150.5 (80–221)	26.51	157.5 (102–198)	25.93	265.500	0.992
INR	1.14 (0.95–1.98)	26.13	1.14 (0.97–2.27)	27.50	252.000	0.772
Alpha-fetoprotein	11.2 (2.14–131)	24.26	26.15 (3.07–110)	32.57	181.000	0.080
Serum creatinine	0.71 (0.4–0.95)	26.39	0.7 (0.52–1.06)	26.79	262.000	0.934
AST	69 (28–224)	24.05	107.5 (38–146)	33.14	173.000	0.055
ALT	66.5 (15–264)	23.95	88 (38–229)	33.43	169.000	0.045 *

INR, international normalized ratio; AST, aspartate aminotransferase; ALT, alanine aminotransferase, * significant.

**Table 4:** Comparison of muscle mass dynamics after SVR between groups with low L3-SMI and normal L3-SMI at baseline.

	Normal L3-SMI (*n* = 19)		Low L3-SMI (n = 33)		Mann-Whitney	
	Median (Range)	Mean Rank	Median (Range)	Mean Rank	U	*p*
L3-SMI difference (numeric)	−3.98 (−16.94 to 23.81)	20.16	−1.3 (−6.94 to 17.34)	30.15	193.000	0.022 *

* significant.

## Data Availability

Data supporting reported results can be provided on request in an electronic format.
